# Fourier transform infrared spectroscopy coupled with machine learning classification for identification of oxidative damage in freeze-dried heart valves

**DOI:** 10.1038/s41598-021-91802-2

**Published:** 2021-06-10

**Authors:** Dejia Liu, Sükrü Caliskan, Bita Rashidfarokhi, Harriëtte Oldenhof, Klaus Jung, Harald Sieme, Andres Hilfiker, Willem F. Wolkers

**Affiliations:** 1 Biostabilization Laboratory, Lower Saxony Centre for Biomedical Engineering, Implant Research and Development, University of Veterinary Medicine Hannover, Stadtfelddamm 34, 30625 Hannover, Germany; 2 Unit for Reproductive Medicine, Clinic for Horses, University of Veterinary Medicine Hannover, Hannover, Germany; 3 Institute for Animal Breeding and Genetics, University of Veterinary Medicine Hannover, Hannover, Germany; 4 Leibniz Research Laboratories for Biotechnology and Artificial Organs, Hannover Medical School, Hannover, Germany

**Keywords:** Biotechnology, Health care, Medical research

## Abstract

Freeze-drying can be used to ensure off-the-shelf availability of decellularized heart valves for cardiovascular surgery. In this study, decellularized porcine aortic heart valves were analyzed by nitroblue tetrazolium (NBT) staining and Fourier transform infrared spectroscopy (FTIR) to identify oxidative damage during freeze-drying and subsequent storage as well as after treatment with H_2_O_2_ and FeCl_3_. NBT staining revealed that sucrose at a concentration of at least 40% (w/v) is needed to prevent oxidative damage during freeze-drying. Dried specimens that were stored at 4 °C depict little to no oxidative damage during storage for up to 2 months. FTIR analysis shows that fresh control, freeze-dried and stored heart valve specimens cannot be distinguished from one another, whereas H_2_O_2_- and FeCl_3_-treated samples could be distinguished in some tissue section. A feed forward artificial neural network model could accurately classify H_2_O_2_ and FeCl_3_ treated samples. However, fresh control, freeze-dried and stored samples could not be distinguished from one another, which implies that these groups are very similar in terms of their biomolecular fingerprints. Taken together, we conclude that sucrose can minimize oxidative damage caused by freeze-drying, and that subsequent dried storage has little effects on the overall biochemical composition of heart valve scaffolds.

## Introduction

Decellularized tissues have been successfully used in a variety of tissue engineering applications^1^. In cardiovascular surgery, decellularized heart valves have been used to replace malfunctioning heart valves^2^. Decellularized allogeneic heart valves have been successfully used for transplantation in patients^3^. Transplanted heart valve scaffolds show cell repopulation and remodeling in vivo, and long-term performance after implantation^4^. In order to ensure off-the-shelf availability in clinical settings, a reliable stock of decellularized heart valves with varying valve and leaflet geometry matching patient requirements is required. Refrigerated storage in a liquid solution is only suitable for short-term storage, whereas cryopreservation and freeze-drying allow long-term storage^5,6^. The advantage of freeze-drying is that freeze-dried specimens do not require complex cold chain logistics during storage and transport.

We have shown that freeze-dried heart valves retain their histoarchitecture and biomechanical properties^7^. In addition, freeze-dried pulmonary heart valves show excellent in vivo clinical performance when transplanted in sheep^8^. For successful freeze-drying, heart valves need to be incubated in highly concentrated sugar solutions for protection against the adverse effects of freeze-drying^9^. It needs to be evaluated, however, whether freeze-dried heart valves are shelf stable. Oxidation reactions are known to be one of the main causes of storage injury in dried specimens. Therefore, oxidative damage of dried heart valves during storage should be minimal, because this may compromise heart valve performance upon transplantation^10^.

Nitroblue tetrazolium (NBT) can be used to visualize and quantify accumulation of reactive oxygen species (ROS) in tissue. The reduction of NBT to formazan upon reacting with ROS/superoxide is evident as a blue precipitate^11,12^. The intensity of tissue blue coloration can be quantified to provide a measure for storage related oxidation processes^13^. Fourier transform infrared spectroscopy (FTIR) can be used to detect changes in biochemical composition associated with oxidative damage in tissues and it has the advantage that it requires minimal sample preparation^9^.

FTIR spectra can be used to identify characteristic molecular group vibrations in complex composite tissue samples. By analyzing specific absorbance bands in infrared spectra obtained from tissues, relative levels of specific tissue compounds including lipids, proteins, and nucleic acids can be detected as well as their in situ aggregational and conformational state^14–16^. The protein amide-I band in infrared spectra of heart valve tissues can be used to quantify relative contents of α-helical, β-sheet and unordered structures, particularly changes in response to processing treatments and/or storage conditions^17,18^.

Infrared spectra of biospecimens comprise of a complex multidimensional dataset of wavenumber versus intensity plots. Principal component analysis (PCA) allows dimension reduction of such a multidimensional dataset in a small set of principal components (PCs). Different treatment groups can be resolved as different clusters in score plots of the PCs and corresponding loadings plots reveal the spectral origin where different sample treatment groups can be distinguished from one another^19^. After PCA, supervised machine learning approaches including feed forward neural network models can be used for a more robust classification based on spectral fingerprinting^20,21^. True and predicted sample classes can be used to create a confusion matrix to depict the classification results.

In this study, decellularized porcine aortic heart valves were analyzed by NBT staining and FTIR to identify oxidative damage during freeze-drying and subsequent storage. Biopsy punches were taken from different tissue locations (i.e., leaflet and artery trunk) and freeze-dried using sucrose as lyoprotectant. Moreover, samples were treated with H_2_O_2_ and FeCl_3_ to artificially provoke oxidative damage. The intensity of tissue blue coloration of NBT-stained specimens was quantified directly after freeze-drying and during storage at 4 and 37 °C. FTIR spectra were taken from artery and leaflet tissue sections, and spectra of treatment groups were analyzed by comparing intensity ratios of characteristic molecular group vibrations, PCA and a feed forward neural network classification model. The results show that 40% sucrose is needed to avoid oxidative damage during the freeze-drying process itself, and that oxidative damage in freeze-dried samples is minimal during storage at 4 °C.

## Results

### ROS formation in heart valve tissues during freeze-drying and subsequent storage

**Figure 1:**
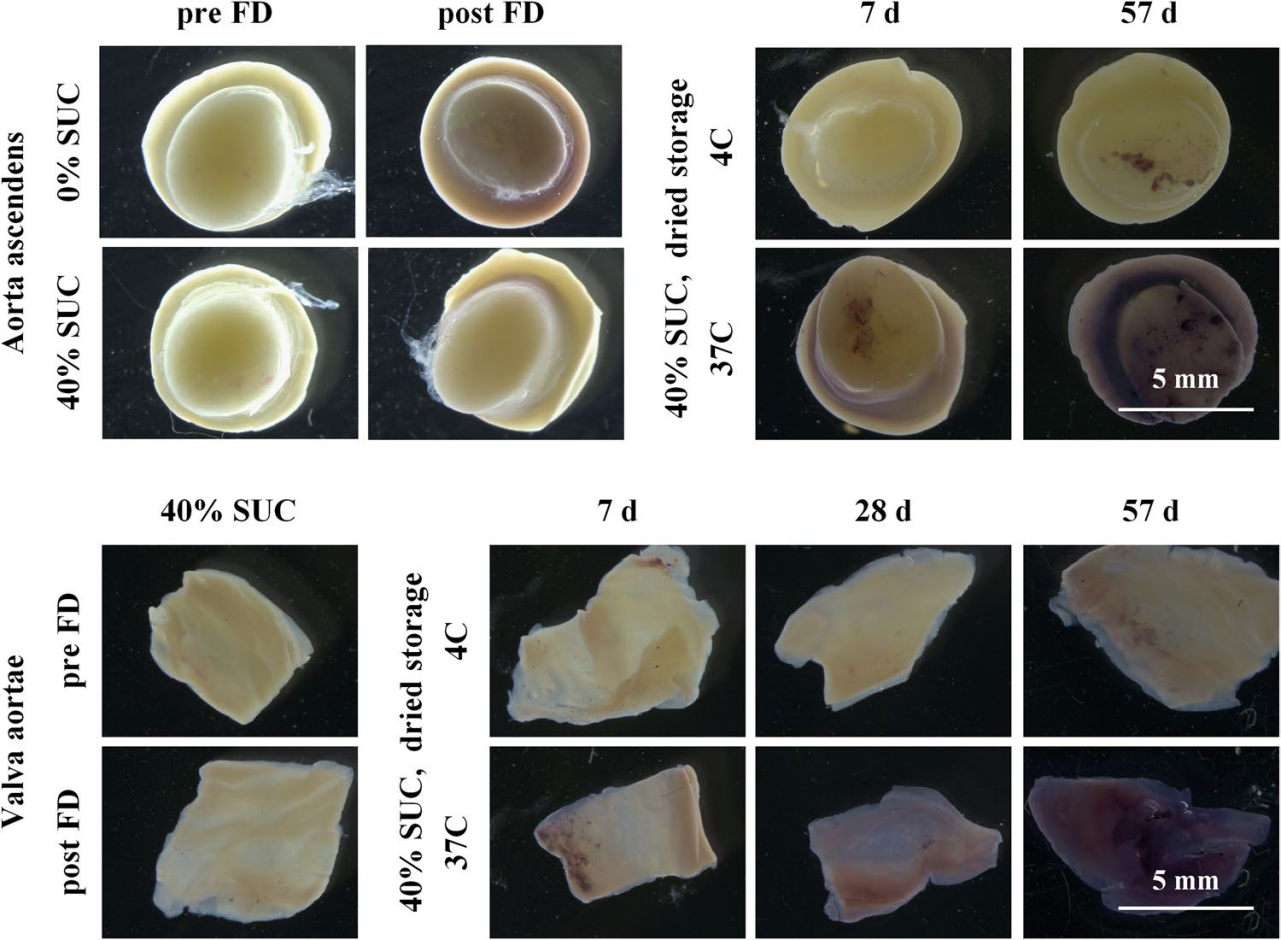
Visualization of accumulation of reactive oxygen species in decellularized and freeze-dried heart valve tissue, by means of NBT-formazan formation. Both artery and leaflet tissues were analyzed, before and after freeze-drying (FD) with 40% sucrose (SUC), and during dried storage at 4 °C and 37 °C for up to 2 months. Microscopic images were collected from fixed samples using similar acquisition settings. Blue/purple staining results from formazan formation, due to NBT reacting with reactive oxygen species.

**Figure 2:**
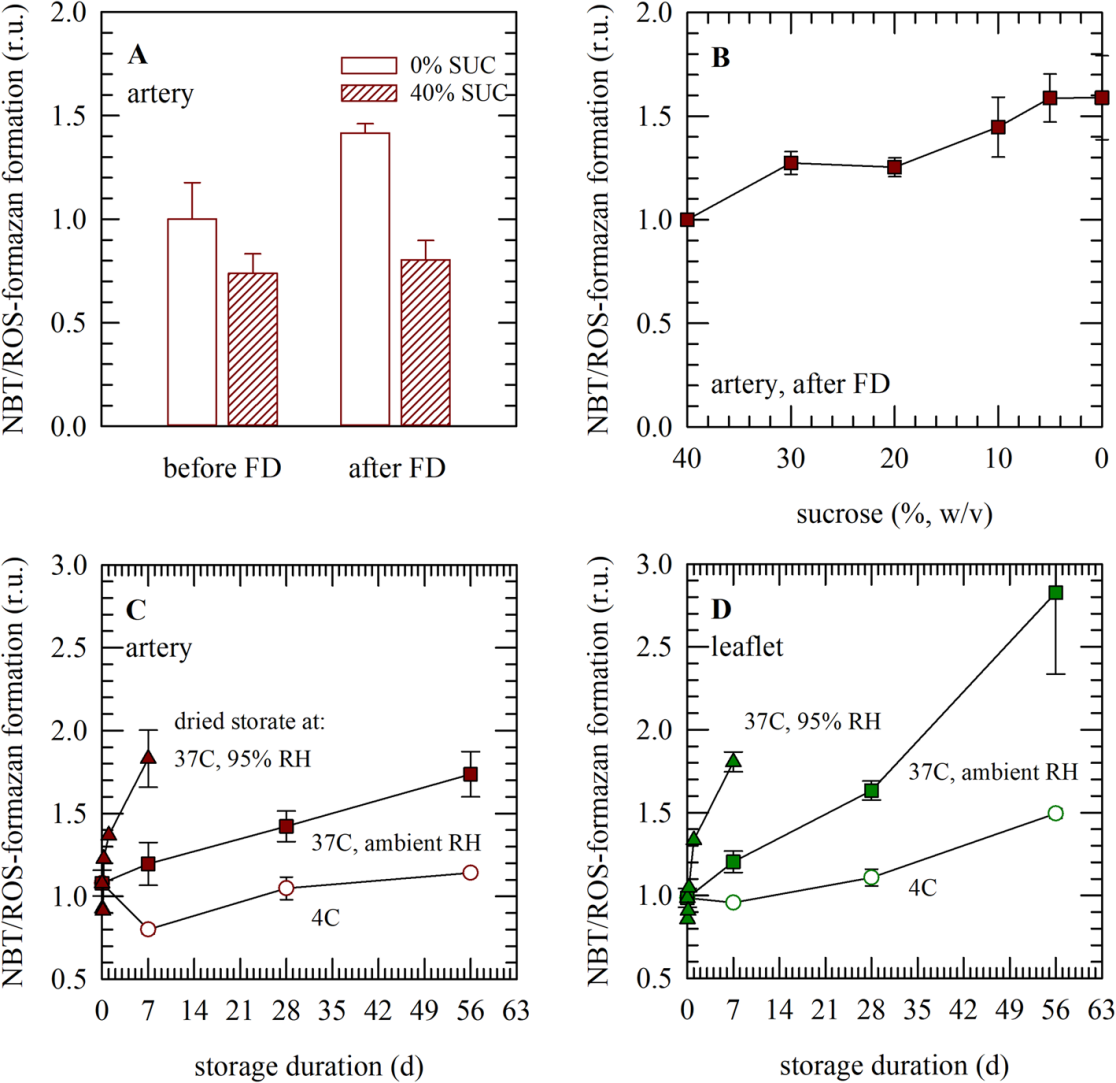
Quantification of accumulation of reactive oxygen species in decellularized and freeze-dried heart valve tissue, analyzed via following NBT-formazan formation. The intensity of blue/purple staining, depicted in Fig. 1, was quantified via image analysis and normalized towards initial/reference values obtained with each data set. This was done for artery tissue, before and directly after freeze-drying with 40% sucrose (**A**) as well as after freeze-drying with sucrose concentrations ranging from 40 to 0% (**B**). Furthermore, both for artery (**C**) and leaflet tissue (**D**), specimens were analyzed during dried storage at 4 °C (circle symbols) and 37 °C (square symbols), under ambient RH, and during storage at high RH at 37 °C (triangle symbols). Mean values ± standard deviations are presented, determined from three to six tissue pieces.

Figure 1 depicts the accumulation of reactive oxygen species in decellularized aortic heart valve tissue sections using the NBT-assay. Both artery and leaflet tissue sections were analyzed before and after freeze-drying with 40% sucrose, and during dried storage at 4 °C and 37 °C for up to 2 months. Blue staining is caused by formazan formation because of NBT reacting with reactive oxygen species. Tissue samples that were freeze-dried in the absence of sucrose stain blue after freeze-drying and rehydration, whereas formazan production is almost entirely prevented when freeze-drying is done in the presence of 40% sucrose. Figure 2 shows the intensity of blue staining in the tissues quantified using ImageJ analysis. Formazan production after freeze-drying shows a dose-dependent decrease with increasing sucrose contents in the freeze-drying formulation. At a sucrose concentration of 40%, the level of NBT staining is similar compared to that prior to freeze-drying. Dried specimens that were stored at 4 °C and ambient relative humidity depict little to no staining during storage for up to 2 months, whereas samples that were stored at 37 °C show a progressive increase in blue staining over time. Storage under high relative humidity conditions resulted in a rapid blue staining, which is already visible after several hours of storage.

### Spectral fingerprinting of freeze-dried heart valve tissues

**Figure 3:**
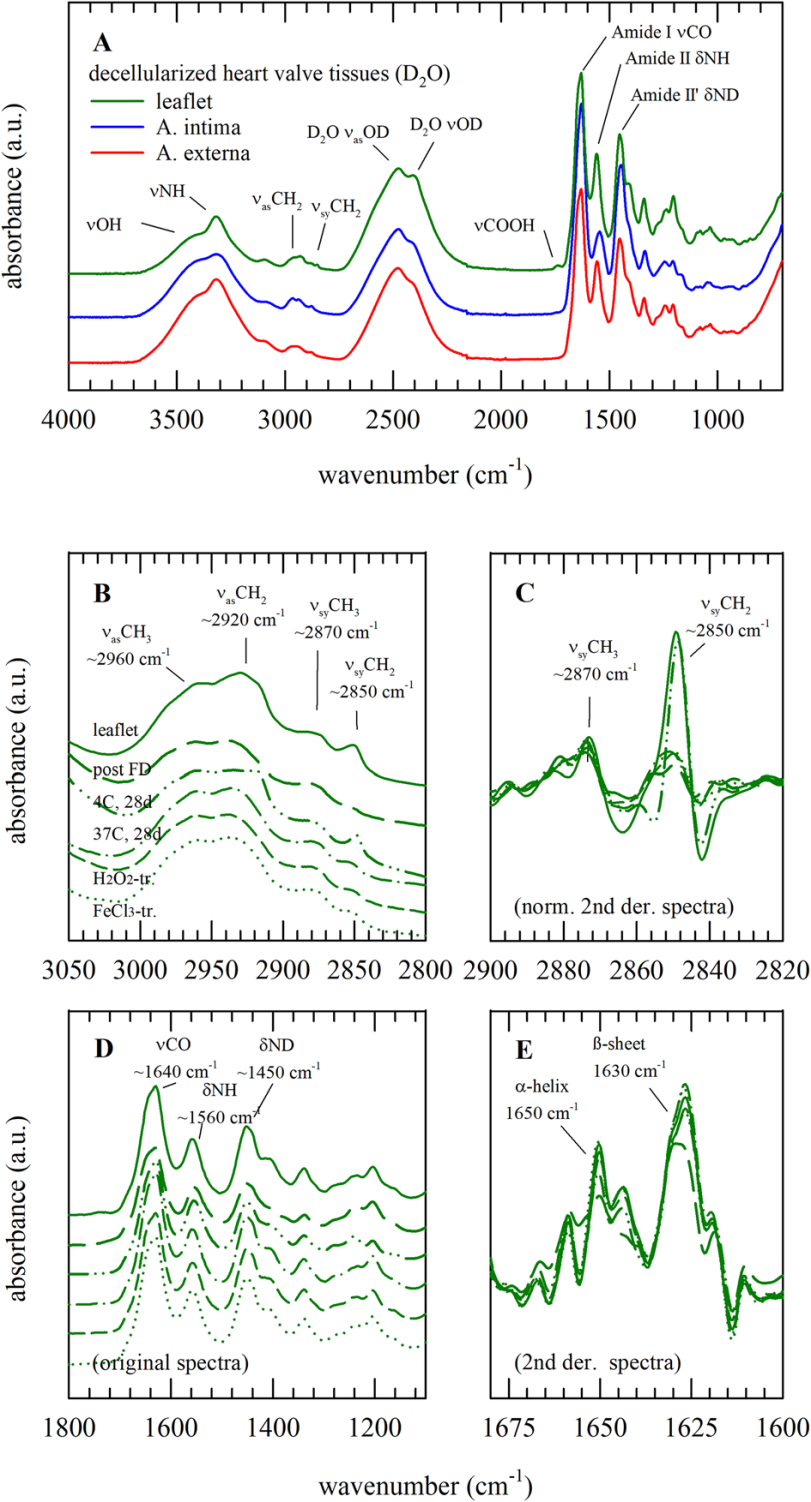
Infrared spectra of different decellularized heart valve tissues (**A**), as well as leaflet tissue exposed to different treatments provoking different degrees of oxidative damage (**B**–**E**). Full original spectra are shown, in which specific absorbance bands are assigned; of leaflet tissue (green lines) and the internal (blue line) and external (red line) artery locations. Furthermore, original (**B**, **D**) and second derivative (**C**, **E**) spectra are presented, for the 3050–2800 cm^−1^ (**B**, **C**) and 1800–1150 cm^−1^ (**D**, **E**) spectral regions containing respectively CH stretching and protein backbone absorbance bands. For the latter (**B**–**E**), spectra are depicted of leaflet tissue before and after freeze-drying, specimens stored for 1 month at 4 °C and 37 °C, and specimens subjected to H_2_O_2_- and FeCl_3_-treatment. Average spectra calculated from six replicates are presented.

Figure 3 shows infrared spectra of decellularized aortic heart valves of leaflet and artery sections subjected to freeze-drying, storage and artificially induced oxidative damage by H_2_O_2_ and FeCl_3_. The different tissue sections, i.e. leaflet, artery externa and artery intima, show characteristic spectral features over the entire spectral range (Fig. 3A). The protein amide I band at ~ 1640 cm^−1^ and the DO stretching band (of the solvent D_2_O) at ~ 2500 cm^−1^ are the most pronounced bands in the spectra. The OH/NH stretching region between 3600 and 3000 cm^−1^ originates from protein OH and NH groups. The CH stretching region between 2800 and 3000 cm^−1^ is dominated by contributions from symmetric and asymmetric CH_2_ and CH_3_ stretching vibrations. This region shows differences among the treatment groups, which are more clearly visible after computing second derivative spectra (Fig. 3B, C). The protein amide-I (νCO) and II (δNH) bands are visible at 1640 and 1560 cm^−1^, respectively. The amide-II’ band (δND) at 1450 cm^−1^ is the result of NH-ND exchange with the solvent (D_2_O), but this band overlaps with the D_2_O scissoring band. The amide-I band can be used to resolve contributions from different types of protein secondary structures, i.e., α-helical and β-sheet structures, which are more clearly visible after computing inverted second derivative spectra (Fig. 3D, E). Qualitative differences are visible in relative contribution of the different types of protein secondary structure among the treatment groups.

**Figure 4:**
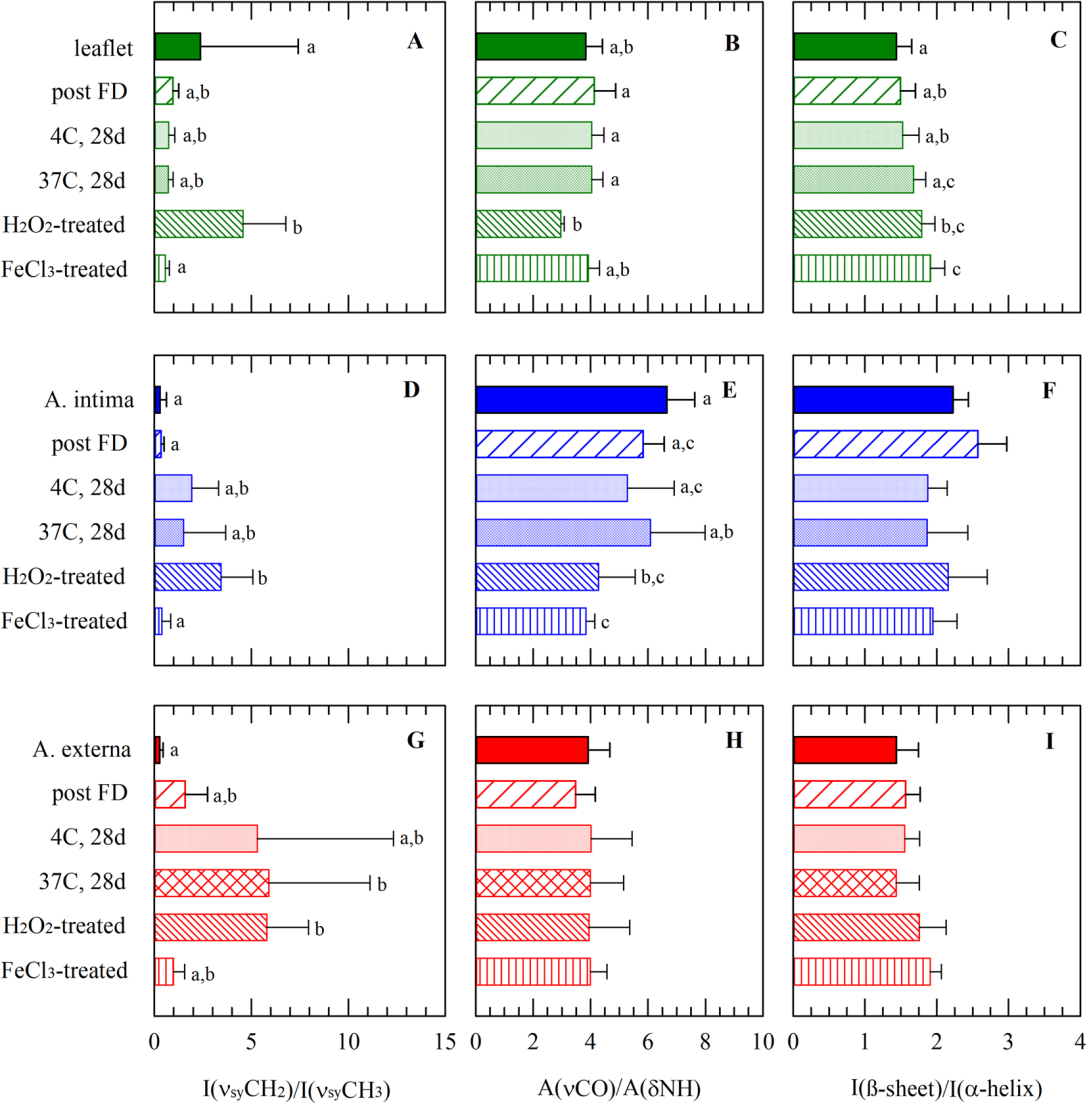
Effects of freeze-drying, dried storage, and induced oxidative damage (i.e., treatment with H_2_O_2_ or FeCl_3_) on infrared spectral fingerprints taken from layers of the aortic leaflet and trunk; expressed as different band ratios. Spectral characteristics were determined for the leaflet (**A**–**C**; green bars), intima (**D**–**F**; blue bars) and externa (**G**–**I**; red bars) artery layer; for specimens before (filled bars) and after freeze-drying (bars with upward diagonal lines), after 1 month storage at 4 °C (dotted bars) and 37 °C (bar with diamonds), as well as specimens exposed to H_2_O_2_ (bar with downward diagonal lines) and FeCl_3_-treatment (bar with horizontal lines). From normalized second derivative spectra in the 2900–2830 cm^−1^ region, band intensity ratios of the symmetric stretching vibrations of the CH_2_ and CH_3_ peaks [I(ν_sy_CH_2_)/ν_sy_CH_3_)] were determined (**A**, **D**, **G**). From original spectra the absorbance band areas of the C = O stretching (at ~ 1640 cm^−1^) and N–H bending (at ~ 1560 cm^−1^) vibration were determined, and their ratio [A(νCO)/A(δNH)] was calculated (**B**, **E**, **H**). From second derivative spectra in the 1700–1600 cm^−1^ region, band intensity ratios of the bands at ~ 1650 and ~ 1630 cm^−1^, representing respectively α-helical and β-sheet structures, [I(β-sheet)/I(α-helix)] were determined (**C**, **F**, **I**). Mean ± standard deviations were calculated from six different spectra, while statistically significant differences are indicated with different letters (*p* < 0.05).

Band ratios of characteristic molecular group vibrations obtained from the spectra were used to further identify effects of freeze-drying, dried storage and induced oxidative damage (Fig. 4). Band intensity ratios of the symmetric stretching vibrations of the CH_2_ and CH_3_ peaks [I(ν_sy_CH_2_)/I(ν_sy_CH_3_)] show statistically significant differences among treatment groups in all tissue sections. H_2_O_2_ treatment increased the [I(ν_sy_CH_2_)/I(ν_sy_CH_3_)], whereas FeCl_3_ treatment did not alter the ratio compared to control, freeze-dried, and stored samples. In the artery externa layer, dried storage at 37 °C increased [I(ν_sy_CH_2_)/I(ν_sy_CH_3_)], whereas the ratio was not affected in the other layers.

The ratio of the absorbance band areas of the C = O stretching (at ~ 1640 cm^−1^) and N–H bending (at ~ 1560 cm^−1^), [A(νCO)/A(δNH)] decreases after H_2_O_2_ treatment in the leaflet and artery intima externa layer, whereas FeCl_3_ treatment only affects this ratio in the artery intima layer. Band intensity ratios of the bands at ~ 1650 and ~ 1630 cm^−1^, representing, respectively, α-helical and β-sheet structures [I(β-sheet)/I(α-helix)] only show significant differences in the leaflet section where the relative proportion of β-sheet structure of FeCl_3_ treated samples is higher compared to the other treatment groups.

### Principal component analysis

**Figure 5:**
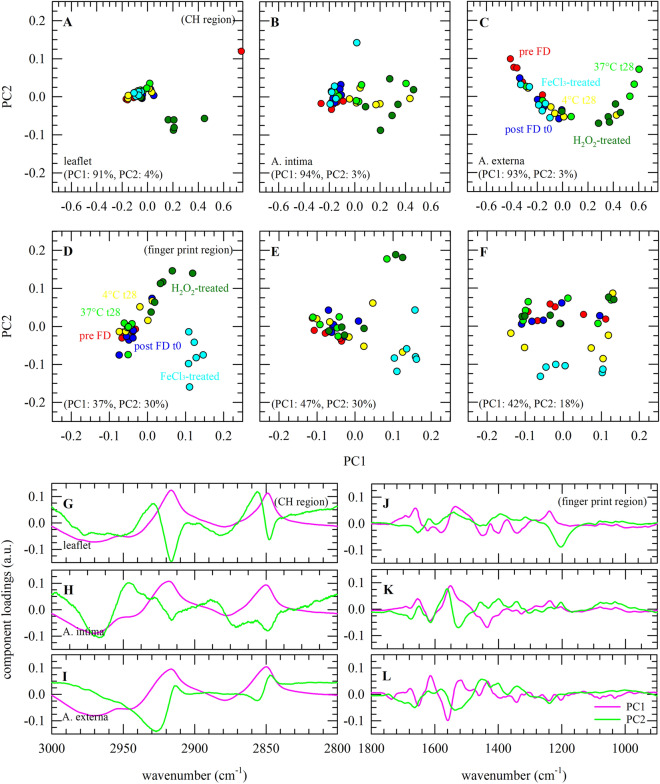
Application of principal component analysis (PCA), for classification of infrared spectra of decellularized heart valve tissues subjected to freeze-drying and dried storage, as well as induced oxidative stress. Analysis was done for the leaflet (**A**, **D**, **G**, **J**), intima (**B**, **E**, **H**, **K**) and externa (**C**, **F**, **I**, **L**) artery layers. PCA was performed for the CH stretching region ranging from 3000 to 2800 cm^−1^ (**A**–**C**, **G**–**I**) and the 1800–900 cm^−1^ spectral range containing protein specific absorbance bands and the fingerprint region (**D**–**F**, **J**–**L**). In all cases specimens were analyzed before (red symbols) and after freeze-drying (dark blue symbols), after 1 month storage at 4 °C (yellow symbols) and 37 °C (light green symbols), as well as specimens exposed to H_2_O_2_ (dark green symbols) and FeCl_3_-treatment (light blue symbols). For both spectral regions, score values of PC1 were plotted versus PC2 (**A**–**F**). In addition, loadings plots of PC1 and PC2 were constructed (**G**–**L**). Measurements and analysis were done using six specimens for each sample group.

Figure 5 shows the results of PCA conducted on infrared spectra of decellularized heart valve tissues subjected to freeze-drying, storage, and induced oxidative stress. Analysis was done for the leaflet and the artery intima and externa layers on both the CH stretching region ranging from 3000 to 2800 cm^−1^ and the 1800–900 cm^−1^ spectral range. Score plots of the first two principal components (PCs) were constructed to evaluate if treatment groups form separate clusters (Fig. 5A–F). PC1 explains most of the observed variance (90–95%) in the CH stretching region and separates H_2_O_2_-treated samples from the other treatment groups. Samples that were stored at 37 °C showed a tendency to cluster in a separate group in the artery layers. The most prominent component loadings for PC1 are seen at ~ 2850 and ~ 2920 cm^−1^ (Fig. 5G–I), which respectively denote the symmetric and asymmetric CH_2_ stretching bands. In the fingerprint region, FeCl_3_ treated samples form separated clusters in all tissue layers, whereas H_2_O_2_ treated samples only form a separate cluster in the leaflet sections. The most prominent component loadings for PC1 in the fingerprint region are seen at ~ 1560 cm^−1^ (Fig. 5J–L), which denotes the NH bending vibration. In the fingerprint region, the relative contribution of PC2 was higher compared to that in the CH stretching region (20–40% vs 1–5%), which likely is related to the fact that the fingerprint region is more complex than the CH stretching region.

### Supervised learning using a feed forward artificial neural network model

**Figure 6:**
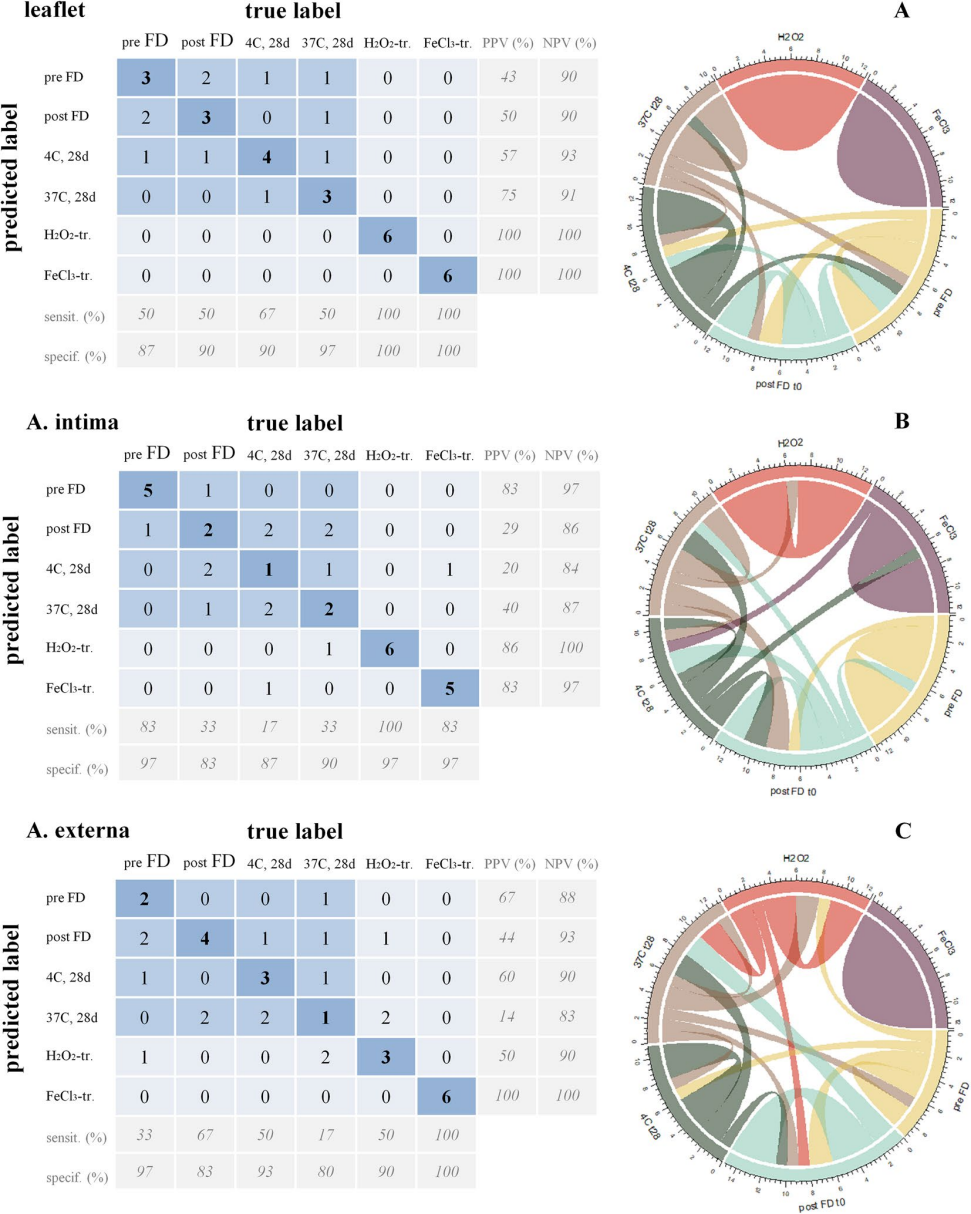
Application of a supervised machine learning approach, by means of a convolutional feed-forward artificial neural network (ANN) model for classification of infrared spectra obtained from decellularized heart valve tissues exposed to freeze-drying and dried storage, as well as induced oxidative stress. Analysis was done for the leaflet, artery intima, and externa layer and the results are presented as confusion matrices (left panels) and chord diagrams (right panels) to demonstrate the interrelationships between different groups. Inside the confusion matrix, discrimination indicates the number of the correctly acquired spectra in the model within each group as the true positive (TP) values. The description of the rows located on the bottom of the confusion matrix indicates the number of observations correctly identified for each predicted class as percentages defined for each group as sensitivity. The description of the rows under the sensitivity displays the proportion of negatives that are correctly identified as specificity. The description of the columns located on the left side of the confusion matrix displays the number of correctly classified observations for each true group as positive predictive value (PPV) and on the right side is negative predictive value (NPV) which means the groups that the test provided gave a negative prediction.

To assess if advanced machine learning approaches can distinguish differently treated heart valve tissues based on their infrared spectra, a feed forward artificial neural network model (ANN) was fitted and evaluated. Figure 6 shows confusion matrices and corresponding chord diagrams obtained with ANN analysis of the infrared spectra in the 1800–900 cm^−1^ spectral range. Analysis was done for the leaflet (A), artery intima (B) and externa (C) layers. If the true label of a sample corresponds to the predicted label, the sample will be counted in the diagonal of the matrix. So, the maximum number on the diagonal for each treatment group is 6 (6 different valves from different animals were included in the analysis). For leaflet tissue subjected to H_2_O_2_ and FeCl_3_, the numbers on the diagonal are 6. The specificity values for spectral discrimination of H_2_O_2_ and FeCl_3_ groups were both 100%, which means that in these cases the established neural network classification approach through interrogated FTIR spectra successfully discriminated H_2_O_2_- and FeCl_3_-treated tissues with 100% positive predictive value and 100% negative predictive value. All samples have been accurately classified by the neural network model. This is also observed for the artery intima layer, the classification model discriminated oxidative damage by H_2_O_2_ and FeCl_3_ tissues with, respectively, 86% and 83% positive predictive value. Whereas for the artery externa layer only the FeCl_3_-treated specimens with 100% positive predictive value were accurately classified. Fresh control, freeze-dried, and stored (4 and 37 °C) samples appear difficult to classify in their corresponding treatment groups. This implies that these groups are very similar in terms of their spectral fingerprints and characteristic molecular group vibrations. This indicates that freeze-drying and subsequent storage has little effects on the overall biochemical composition of the scaffolds. The loss-and-accuracy functions of the neural network model are depicted in Supplementary Fig. 1. The accuracy of the model approaches 1 in all cases. For comparison, linear discriminant analysis (LDA) was also used to analyze the data. The confusion matrices obtained by LDA (Supplementary Fig. 2) are similar compared to those obtained by the ANN model, indicating LDA can also be used for classification in this case.

## Discussion

In order to ensure shelf stability of freeze-dried heart valves that are intended to be used in cardiovascular surgery, damage during processing and storage should be minimized. NBT staining and FTIR were used here to identify oxidative damage in freeze-dried heart valves. Oxidative damage can occur during the freeze-drying process itself and can further accumulate during storage under suboptimal conditions. Tissues that are freeze-dried in the absence of sucrose exhibit oxidative damage, whereas oxidative damage is almost entirely prevented when freeze-drying is done in the presence of 40% sucrose. Oxidative damage progressively decreases with increasing sucrose contents in the freeze-drying formulation, and at 40% sucrose, the freeze-drying process does not cause measurable oxidative damage. The oxidative damage (NBT reduction) that is caused by freeze-drying in the absence of sucrose can likely be attributed to the formation of pores in the tissue, which are the result of ice crystals disrupting the tissue histoarchitecture^7^. Sucrose diminishes pore formation in freeze-dried heart valves in a dose-dependent manner, and 40% sucrose is needed to entirely prevent pore formation^9^. Interestingly, this dose-dependent decrease in tissue porosity with increasing sucrose concentration coincides with the decrease in oxidative damage (blue staining) that was found here.

We previously used NBT staining to detect oxidative damage in ovarian cortex tissues stored in liquid^13^. In liquid, NBT reduction in these tissues was detected within several hours, and could only be effectively stopped at low subzero temperatures. The absence of water slows down the rate of chemical reactions including oxidation reactions^22,23^. Dried heart valve specimens that were stored at 4 °C depict little to no staining during storage for up to 2 months. Samples that were stored at 37 °C show signs of oxidative damage already after 1 week storage. Storage at elevated temperatures results in faster molecular motions, which increase the rate of chemical reactions.

If freeze-dried samples are stored under high relative humidity conditions, oxidative damage is visible within hours. Water has a plasticizing effect on the amorphous state of freeze-dried samples^24^ because absorption of water by dried samples will increase molecular mobility^25^. Heart valves, freeze-dried with sucrose, have a glass transition temperature (Tg) at around 45 °C^7^, which means they are in a protective glassy state at room temperature. The Tg of sucrose decreases with increasing water contents^26^, and likewise the Tg of freeze-dried heart valves also decreases with increasing water content^7^. If the glass transition temperature drops below ambient temperatures, the sample is basically in a liquid-like state and oxidation reactions occur at a much faster rate.

FTIR spectroscopy is a highly discriminatory technique for complex samples as it is based on characteristic absorption patterns of endogenous molecules providing unique spectral fingerprints^27,28^. We have previously shown that FTIR spectroscopy is an effective method to identify oxidative damage, or lack thereof, in tissue^18^. Band intensity ratios of the symmetric stretching vibrations of the CH_2_ and CH_3_ peaks show differences among treatment groups in all tissue sections. The ratio of the absorbance band areas of the C=O stretching (at ~ 1640 cm^−1^) and N–H bending (at ~ 1560 cm^−1^), [A(νCO)/A(δNH)] decreases after H_2_O_2_- and FeCl_3_-treatment in the artery intima layer. The overall protein secondary structure seems not to be affected by freeze-drying and storage, as is evident from intensity ratios of the bands at ~ 1650 and ~ 1630 cm^−1^ in the amide-I region. Only FeCl_3_ treatment appears to affect the overall protein secondary structure of the leaflets, whereas the overall protein secondary structure of samples that were stored at 37 °C appears to be well preserved.

Comparing band intensities in FTIR spectra does not take simultaneous changes in other group vibrations into account. PCA can be used to explore similarities and differences in spectral features based on the entire spectrum (or in selected regions). The region from 1800 to 900 cm^−1^ was used for PCA, because this gave a somewhat better cluster separation due to the exclusion of regions with little or no molecular vibrations. FeCl_3_-treated tissue was visible as a separate cluster in all three microstructures. Control, freeze-dried and stored samples do not form separate clusters, indicating these groups are similar in terms of biochemical composition. In the 3000–2800 cm^−1^ spectral range, the leaflet and artery intima that were subjected to H_2_O_2_-treated oxidative damage formed separate clusters in PCA score plots, whereas FeCl_3_ treated tissue could not be identified as a separate cluster. The main component loadings of PC1 correspond to symmetric and asymmetric CH_2_ stretching vibrations^18^.

Machine learning and deep learning are increasingly being used to analyze vibrational spectral data sets for diagnostic or classification purposes^21,27^. Neural network classification models can be used as feature extractor method, because of their ability to learn complex features directly from spectral data^28^. FTIR combined with artificial neural network algorithms has been used to discriminate mycelia of fungal strains^29^, to identify different bacterial strains^30^, to identify the major cellular and acellular constituents in (tumor) tissues^31^, and to investigate quantitative changes of biomarkers in plasma for disease diagnostics^32^. This is related to the advantage of neural networks in generating decision boundaries in statistical models and the inherently nonlinear predictive ability of ANNs. It was demonstrated here that a neural network classification model, trained with FTIR spectra from different heart valve samples, can be used to classify heart valves subjected to various treatments. In this case, the neural network model is used to extract spectral differences caused by alterations in tissue biochemical composition resulting from oxidative damage. Both H_2_O_2_- and FeCl_3_-treated tissues were accurately classified using the neural network model. Fresh control, freeze-dried, and stored (4 and 37 °C) samples, however, could not be classified, indicating that these samples have similar characteristic spectral features. This indicates that freeze-drying and subsequent storage does not have major effects on the overall biochemical composition of decellularized heart valves. The advantage of combining FTIR spectroscopy with machine learning is that oxidative damage can be evaluated using standard classifier sets of samples with defined levels of oxidative damage. FTIR combined with machine learning could thus be used for quality assurance of (freeze-dried) heart valve implants stored in biobanks that are meant for clinical use. FTIR does not require labeling, and data collection is faster compared to histological assessment, which requires labeling and is time-consuming. ANN and LDA provided similar classification results. It should be noted that LDA, like PCA, as a linear discriminant method, has its limitations. Nonlinear relationships are quite common in many practical cases. Beam scattering, system alignment, accessory effects, system optic, sample inhomogeneity, and changes in geometry between samples and standards can all cause deviations from Beer's law in FTIR analysis^33^. In addition when the sample classification information of LDA depends on the variance rather than the mean, dimension reduction cannot provide good performance. In this case, an ANN-based classification approach with both linear and nonlinear modeling capabilities can provide a solution.

In conclusion, it is shown here that sucrose minimizes the extent of oxidative damage in decellularized heart valves caused by freeze-drying in a dose-dependent manner. A 40% (w/v) sucrose solution is needed to avoid oxidative damage during freeze-drying. Dried storage at standard refrigeration temperatures is sufficient to minimize oxidation reactions. If dried specimens are stored at elevated temperatures, or under high relative humidity conditions, they show increasing levels of oxidation damage over time. Oxidation reactions during storage, however, do not result in major changes in the overall biochemical composition as became evident from FTIR combined with machine learning. Samples that are stored at elevated temperatures cannot be distinguished from control and freeze-dried valves not subjected to storage.

## Materials and methods

### Heart valve decellularization and freeze-drying

Fresh porcine hearts (n = 9) were obtained from a local slaughterhouse and transported to the lab in phosphate buffered saline (PBS; 137 mM NaCl, 27 mM KCl, 10 mM Na_2_HPO_4_, 1.8 mM KH_2_PO_4_, pH 7.4) supplemented with 1% (v/v) penicillin. Aortic valves were excised, and washed in 7.5% (w/v) iodine solution and PBS, 5 min each. Decellularization was performed as previously described^9^. In brief, valves were treated with 0.5% (v/v) Triton X-100 for 24 h while shaking, followed by 24 h treatment with 0.5% (w/v) SDS, and 24 h washing with deionized water. Detergent solutions were replaced by fresh solutions every 12 h. Finally, valves were subjected to washing in distilled water (2 × 12 h) and PBS (2 × 12 h), after which they were stored in PBS at 4 °C until use.

Cylindrically shaped tissue punches (5 mm diameter) were cut from the artery as well as valve regions using a biopsy punch. Prior to freeze-drying, specimens were maintained in PBS or incubated for 4 h at 22 °C in PBS supplemented with 0–40% (w/v) sucrose. Incubation of the tissues in sucrose solution was done on an orbital shaker, while durations (4 h) were estimated based on previous measurements on sucrose diffusion into heart valve tissues^17^. After sucrose loading, tissue pieces were transferred to petri dishes and placed in a − 80 °C freezer and kept there for 3 h. The samples were freeze-dried using an Alpha 2–4 LSC plus freeze-dryer (Martin Christ Gefriertrocknungsanlagen GmbH, Osterode am Harz Germany), using a vacuum pressure of 0.015 mbar, and a condenser temperature of − 85 °C. Dried valves were stored in sealed petri dishes. The water content after freeze-drying is 0.1 g H_2_O/g dry weight, which was determine by comparing fresh (i.e. after freeze-drying) and dry weights. To determine dry weights, samples were placed overnight in an incubator set at 80 °C^7^. Dried tissue specimens were rehydrated by adding water, followed by washing in PBS (3 × 30 min) to remove sucrose.

### Quantification of ROS/NBT-formazan formation in tissues, using image analysis

Nitroblue tetrazolium (NBT) was used to visualize and quantify accumulation of reactive oxygen species (ROS) in tissues, during dried storage as previously described^13^. NBT forms formazan upon reacting with ROS/superoxide which is evident as a blue/purple precipitate. Prior to freeze-drying, tissue was incubated 4 h at 22 °C in PBS supplemented with 0–40% sucrose and 0.1% (w/v) NBT. After rehydration, specimens were transferred into fixation solution (4% paraformaldehyde in PBS), and stored at 4 °C.

For image analysis, micrographs were collected for each specimen using a stereomicroscope with camera setup. Images were taken using a similar magnification and exposure time. Using ImageJ software (National Institutes of Health, Bethesda, MD, USA), micrographs were converted to 8-bit images. The entire area of the tissue was selected in the images, and a global calibration was set. The data were converted into gray scale values, and the mean gray scale intensity of the selected regions was derived as a measure for the intensity of blue coloration (i.e. NBT being converted to formazan upon reacting with ROS). Each treatment was performed in triplicate, i.e. three different tissue biopsies obtained from different animals.

### Fourier transform infrared (FTIR) spectroscopic measurements

Infrared spectra were recorded using a Nicolet iS5 FTIR spectrometer (Thermo-Fisher), equipped with a triglycine sulfate detector and an attenuated total reflection (ATR) accessory, with pressure arm and a diamond/ZnSe crystal. The following spectra acquisition parameters were used: 4 cm^−1^ resolution, 16 co-added interferograms, and 4000–525 cm^−1^ wavenumber range. An automatic CO_2_/H_2_O vapor correction algorithm was applied to subtract interference from atmospheric CO_2_ and H_2_O vapor.

Tissue samples in PBS with 40% sucrose were exposed to freeze-drying and subjected to dry storage at 4 and 37 °C for up to 3 months. In addition, decellularized tissues not exposed to freeze-drying were treated with H_2_O_2_ and FeCl_3_ to provoke oxidative damage. This was done by incubating tissue punches in solutions containing 3% (v/v) H_2_O_2_ or 67 mM FeCl_3_ for 24 h incubation at 22 °C. Hydrated and rehydrated tissue samples were transferred into D_2_O for 30 min prior to analysis. Tissue samples were pressed on the ATR crystal using the pressure arm. For each treatment, six specimens were investigated.

### Spectral analysis: band ratios of characteristic molecular group vibrations

Spectral analysis was conducted in different spectral regions, including the 3000–2800 cm^−1^ region containing CH stretching vibrations, the 1700–1600 cm^−1^ containing protein C = O stretching bands, and the 1600–1500 cm^−1^ containing protein N–H bending bands (not exchanged to N–D). In order to better resolve specific absorbance bands, second derivative spectra were calculated using the Savitzky-Golay method with a 21-point smoothing factor. Hydrated and rehydrated samples were analyzed in D_2_O to avoid interference from H_2_O absorbance bands.

The 3000–2800 cm^−1^ spectral region contains absorbance bands of symmetric stretching vibrations of CH_3_ and CH_2_ groups, at ~ 2870 and ~ 2850 cm^−1^, respectively. The band intensity ratio of [I(νCH_2_)/I(νCH_3_)] was obtained from inverted second derivative spectra. The 1700–1600 cm^−1^ spectral region comprises absorbance bands resulting from α-helical (~ 1650 cm^−1^) and β-sheet (~ 1630 cm^−1^) structures. The band intensity ratio [I(β-sheet)/I(α-helical)] obtained from inverted second derivative spectra, was taken as a measure for changes in the overall protein secondary structure caused by any of the treatments. In addition, the band areas of the amide-I (at ~ 1640 cm^−1^) and amide-II (at ~ 1560 cm^−1^) bands were determined from original spectra, and their ratio was calculated [A(νCO)/A(δNH)]. This was done by determining the baseline corrected areas between 1709 and 1591 cm^−1^ for the amide-I band and between 1592 and 1520 cm^−1^ for the amide II band.

Minimally 6 samples obtained from different heart valves (i.e. from different animals) were measured by FTIR for each treatment group. Spectra were collected from both leaflet and artery sections of the valves and for each treatment, mean ± standard deviations (n = 6) were calculated. One-way analysis of variance (ANOVA) followed by Tukey's multiple comparisons tests were conducted to determine statistically significant differences (*p* ≤ 0.05) among treatment groups.

### Spectral analysis: principal component analysis and machine learning model

Spectra that were collected from both leaflet and artery sections of the valves (six samples obtained from different animals) were used for principal component analysis (PCA) and machine learning analysis. PCA was performed in different spectral regions, including the 3000–2800 and 1800–900 cm^−1^ regions. Prior to PCA, these spectral regions were vector normalized as previously described^13^. PCA was carried out using standard functionality of the statistical programming environment R (version 4.0.3)^34^. PCA allows dimension reduction of a multidimensional dataset into a small set of principal components (PCs) that still explain a portion of variability in a data set. Score plots of the PCs (typically PC2 vs PC1) were used to determine if treatment groups can be resolved as separate clusters. In addition, corresponding loadings plots were constructed to reveal the spectral origin(s) of the differentiation between clusters representing different groups/treatments. Principal component analysis was followed by linear discriminant analysis (LDA) as previously described^35^. In LDA, the ratio of the between-cluster variance to the within-cluster variance is maximized in forming the clusters.

An artificial neural network was used as a machine learning approach for data analysis. Artificial neural networks (ANNs) are inspired by the structure of the nervous system, and are made of artificial neurons arranged in several layers: an input layer, hidden layer(s) (usually one to three), and an output layer. The basic core unit of a neural network consists of a finite number of neurons, the function of which is to find the inner product of the input vector and the weight vector, and to obtain a scalar result through a nonlinear transfer function. Input data, which in this case are vector normalized absorbance intensity values in a given spectral range of an IR spectrum of a sample, are activated by the neurons in the input layer, and then transferred to the hidden layers for weight update of the loss function and feature extraction. Finally, the output layer is used to dock with the hidden layer and output of the modeling results. The weights are adjusted to correct the response to the neuronal stimulation of the different hidden layers, and the resulting output. Vector normalized spectra in the 1800–900 cm^−1^ region, were subjected to supervised machine learning using the R-package ‘keras’^36^ running on an octa-core CPU laptop using single NVIDIA GeForce GTX 1650 graphics card. The open-source machine learning framework, TensorFlow, has been employed for constructing a feed forward artificial neural network (ANN), algorithm training and evaluation. Leave-one-out cross-validation (LOOCV) was applied to evaluate the performance of the ANN. The ANN consisted of an input layer, two hidden layers and an output layer. Vector normalized FTIR spectra were used to activate the network through the input layer, and the two hidden layers were used to extract the essential spectral features with a rectified linear unit (ReLU) as the active function, which returns 0 if it receives any negative input, but for any positive value x it returns that value back. The hidden layer consisted of two layers, each with 128 neurons. The final output layer was applied to control the probability of the classification using the Softmax activation function, which converts a vector of numbers into a vector of probabilities. Each value in the output of the Softmax function is interpreted as the probability of membership for each class.

The ReLU is defined as:

1 $$f\left( x \right) = \max \left( {0,x} \right)$$

The Softmax activation function is defined as:

2 $$\sigma \left( x \right)_{j} = \frac{{e^{{z_{j} }} }}{{\mathop \sum \nolimits_{k = 1}^{K} e^{{z_{k} }} }}\;for\quad j = 1, \ldots , K$$

The process was optimized using the Adam algorithm. Loss and accuracy function were added to evaluate the ANN model and monitor the error during the training process. The learning cycle updates the weights of the output layer. The final output values are probability values between 0 and 1. A confusion matrix was used for classification of true and predicted sample labels. Chord diagrams, as derived from the confusion matrices, were prepared using R^34^ to display the misclassification and correlation of the test set. Model performances were reported by the overall accuracy.

In addition, the following terms have been determined for the classification merits of the model: (i) True Positive (TP), which corresponds to the number of observations originally positive that are also defined as positive; (ii) True Negative (TN), which corresponds to the number of observations initially negative that are also defined as negative; (iii) False Positive (FP), corresponding to the number of observations originally negative but positively graded, and (iv) False Negative (FN), corresponding to the number of observations originally positive but negatively defined. Sensitivity, specificity as well as positive and negative predictive value for each individual class was extracted from the confusion matrices by regarding positive and negative classification frequencies in terms of each class versus all other classes. Sensitivity, specificity, positive predictive value (PPV), and negative predicted value (NPV) are defined as:

3 $${\text{Sensitivity}} = \frac{{{\text{TP}}}}{{{\text{TP}} + {\text{FN}}}} \times 100,$$

4 $${\text{Specificity}} = \frac{{{\text{TN}}}}{{{\text{TN}} + {\text{FP}}}} \times 100,$$

5 $${\text{Positive}}\;{\text{predictive}}\;{\text{value}} = \frac{{{\text{TP}}}}{{{\text{TP}} + {\text{FP}}}} \times 100,$$

6 $${\text{Negative}}\;{\text{predictive}}\;{\text{value}} = \frac{{{\text{TN}}}}{{{\text{TN}} + {\text{FN}}}} \times 100.$$

Accuracy and the other measures of performance were accompanied by 95% confidence intervals^37^.

## Supplementary Information

Supplementary Information 1.
