# Supplementary material for: Fourier transform infrared spectroscopy coupled with machine learning classification for identification of oxidative damage in freeze-dried heart valves
Source: Sci Rep. 2021 Jun 10;11:12299. doi: 10.1038/s41598-021-91802-2 (PMC8192956; doi:10.1038/s41598-021-91802-2)
Supplement: Supplementary file 1 — Supplementary Information 1. [file 41598_2021_91802_MOESM1_ESM.docx]

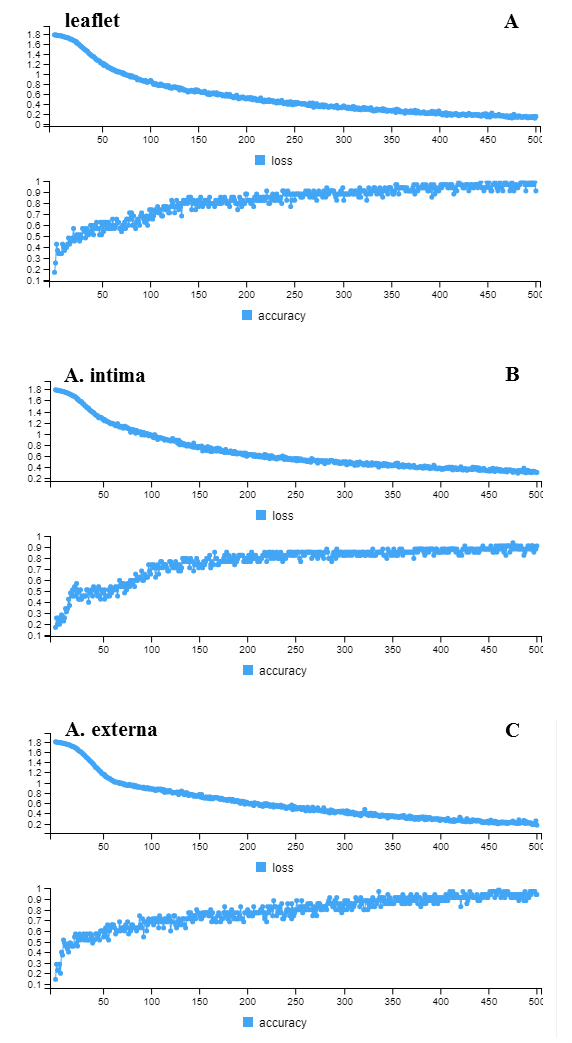


**Supplemental Figure 1.** Loss-and-accuracy functions of the ANN model that was used to classify differently treated decellularized heart valve tissues based on infrared spectra in the 1800‒900 cm^‒1^ spectral range. The numbers on the x-axis reflect the number of iterations, and the numbers on the y-axis, the loss values in arbitrary units and corresponding accuracy loss values between 0 and 1.


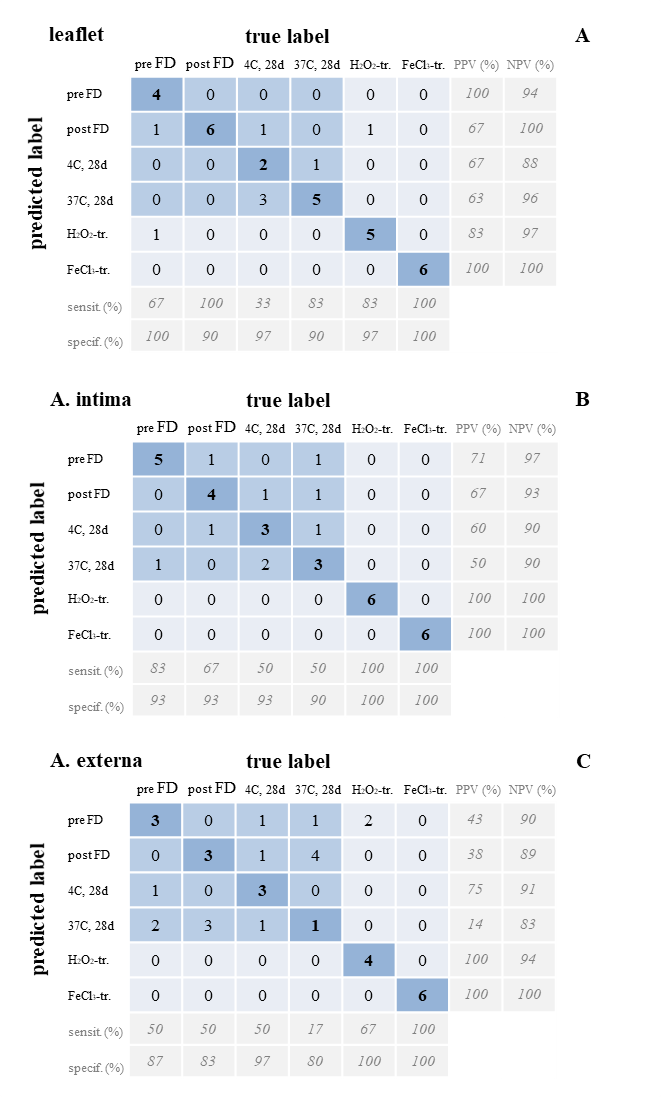


**Supplemental Figure 2.** Application of LDA for classification of infrared spectra obtained from decellularized heart valve tissues exposed to freeze-drying and dried storage, as well as induced oxidative stress. Analysis was done for the leaflet, artery intima, and externa layer and the results are presented as confusion matrices to demonstrate the interrelationships between different groups. Inside the confusion matrix, discrimination indicates the number of the correctly acquired spectra in the model within each group as the true positive (TP) values. The description of the rows located on the bottom of the confusion matrix indicates the number of observations correctly identified for each predicted class as percentages defined for each group as sensitivity. The description of the rows under the sensitivity displays the proportion of negatives that are correctly identified as specificity. The description of the columns located on the left side of the confusion matrix displays the number of correctly classified observations for each true group as positive predictive value (PPV) and on the right side is negative predictive value (NPV) which means the groups that the test provided gave a negative prediction.
